# Roles of Cold Plasma Treatment on the Functional Properties of Plant‐Based Proteins

**DOI:** 10.1155/ijfo/2354428

**Published:** 2026-08-17

**Authors:** Aishat O. Adejoh, Linda A. Kpomblekou, Flora G. C. Ekezie, Titilayo O. Bamidele, Alberta N. A. Aryee, John O. Onuh

**Affiliations:** ^1^ Department of Food and Nutritional Sciences, Tuskegee University, Tuskegee, Alabama, USA, tuskegee.edu; ^2^ SGS North America, Bloomfield, New Jersey, USA; ^3^ Department of Biochemistry, Nasarawa State University, Keffi, Nasarawa, Nigeria, nsukonline.net; ^4^ Department of Human Ecology, Delaware State University, Dover, Delaware, USA, desu.edu

**Keywords:** cold plasma, nonthermal processing, plant-based proteins, protein functionality, structural modification

## Abstract

The growing demand for sustainable, ethical, and healthier foods has generated interest in plant‐based proteins as alternatives to animal proteins. Although promising, these proteins often face limitations due to inherent characteristics that restrict their application diverse food products. These include low solubility, foaming, emulsification, and gelation, which collectively impede their utilization in food systems. To overcome this, cold plasma, a nonthermal and eco‐friendly technology, has recently emerged as a promising strategy to enhance protein functionality without compromising nutritional quality. This review examines the mechanisms, functional outcomes, and food applications cold plasma in plant‐based proteins, with focus on comparison with conventional and emerging protein modification technologies. Rigorous literature search was conducted to obtain relevant information from databases used to develop the review. The results revealed that plasma treatments usually generate reactive oxygen and nitrogen species, ultraviolet photons, and charged particles that can unfold protein structures, promote cross‐linking, and alter surface properties like charge and hydrophobicity. These modifications improve hydration, dispersion, and stability, and enhance performances in emulsions, foams, and gels in plant protein products. Cold plasma offers advantages in terms of energy efficiency and versatility, despite challenges of reproducibility, oxidation, scalability, and consumer acceptance. Combining cold plasma with other processing techniques like ultrasound or fermentation, while applying advanced omics approaches could offer better understanding and open opportunities for food applications. Overall, cold plasma offers a valuable approach to enhance functionality of plant proteins, with strong potential to support innovation, sustainability, and nutrition in the food industry.

## 1. Introduction

### 1.1. Overview of the Review

Plant‐based proteins are becoming prominent owing to their enormous health benefits, lower impacts on the environment, and ethical consideration compared with proteins of animal sources [1]. They are abundant and capable of meeting human protein requirements and provide various essential amino acids including methionine, lysine, and several others. However, some proteins of plant sources may be low in some particular amino acids such as lysine in cereals and methionine in legumes, and consequently possess lower digestibility and potential to support tissue growth and physiological development in comparison with proteins of animal sources [2]. Soy protein is distinct due to its high protein quality and digestibility, which closely matches those from animal sources. Other plant sources of proteins including peas and chickpeas are also being utilized in the food system for their excellent nutritional and functional properties [3]. Plant‐based proteins are employed in the production of several food products such as meat analogues, beverages, baked foods, and pastas because of their functional properties including hydration, emulsification, foaming, and gelation capacities [4].

Nonthermal technologies such as ultrasound, cold atmospheric plasma, high‐pressure processing (HPP), and nonthermal microwave processing are emerging innovations for the modification of proteins in the food industry [5, 6]. They can alter protein structure and functionality. The technologies are devoid of challenges associated with the conventional thermal processing methods like aggregation, excess protein unfolding, and loss of nutritional qualities, while meeting the needs of consumers of minimally processed foods [7]. Nonthermal processing has shown great prospects in improving the hydration, solubility, emulsification, gelation foaming, and digestibility of plant‐based proteins [8]. The effectiveness of these modifications depends on several factors such as protein types and processing factors. Although these innovative nonthermal methods are promising, methods like cold plasma are still in their early developmental stages of application for industrial use and may require further knowledge to fully comprehend their mechanisms and optimize their use for industrial purposes [9].

Proteins are known for their numerous functional properties that are important for their biological roles and applications in the food industry. Some of these functional properties include water‐holding capacity (WHC) and oil‐holding capacity (OHC), hydration, solubility, emulsification, foaming, gelation, viscosity, and texturizing properties, all of which influence the processing, texture, viscosity, and sensory properties of food products [10–12]. These properties are governed by the molecular structure of the proteins such as the primary, secondary, and tertiary arrangements. Physicochemical properties such as hydrophobicity, charge, and ability to interact with other molecules also play a key role in the food industry [13]. They are affected by extraction and processing methods, which may lower or improve the behavior of protein including foaming and solubility properties, and hence can be modified through some novel techniques to improve performance in the food systems [14].

Cold plasma is perceived as a sustainable innovation for the processing of foods owing to its ability to efficiently sterilize and remove contaminants in foods without passing through any form of thermal or chemical treatment, thereby preserving nutritional and sensory properties of the food [15]. The method produces reactive gases that are capable of inactivating microorganisms, breaking down toxins and pesticides as well as improving shelf life while utilizing low amounts of energy and producing little emission in comparison with other methods [16, 17]. It is capable of improving the functional properties of food proteins, enhancing extraction yields of bioactive compounds, and improving safety and barrier performance of food packaging material [18]. It is versatile and allows its application in most stages of food production such as decontamination, inactivation of enzymes, and waste valorization, supporting circular economic principles [19–21]. However, little attention has been given to integrating the basis of plasma‐induced structural changes in plant proteins with their functional consequences in comparison with other conventional methods. More so, differences in how major plant protein sources respond, and in particular, cereal and legume proteins, are not well understood. The objective of this review, therefore, is to critically examine the mechanisms, functional outcomes, and food applications of cold plasma in plant‐based proteins, with a focus on comparisons with conventional and emerging protein modification technologies. This will provide the framework for assessing its efficiency and specificity as well as its applications in the food industry.

## 2. Methodology and Data Collection

### 2.1. Literature Search

The PRISMA 2020 flow guideline for systematic reviews, developed by the European Food Safety Authority (2010), was followed for relevant literature search for this review. The searches were carried out from two major online databases: Google Scholar and ScienceDirect, with focus on those published between 2020 and 2026. In addition, the search terms and other selection criteria were modified during the search to align with the research objective.

### 2.2. Search Criteria and Search Strings

The inclusion and exclusion criteria for article selection were established with the use of advanced search functions. Search terms like “plant protein,” “cold plasma,” “protein functionality,” “nonthermal processing,” structural modification were used. In the advanced search, “AND” and “OR” were used to filter the results, including articles that contain these words anywhere. Using search strings, complete text articles, documents, books, reviews, and systematic reviews were added. No filter was applied for article type, publication source, or authors, and citations were further assessed on their relevance to the study.

### 2.3. Search Process

After the choice of publications according to the key search phrases, assessment of abstracts, titles, and keywords helped exclude duplicate and inapplicable works. Publications that matched the selection requirements were included after a comprehensive assessment both “relevant” and “possibly relevant” categories of references by reviewing their full texts. This review covers all studies that meet the selection criteria.

### 2.4. Classification of Relevant Publications

The papers were reviewed to obtain relevant information that was then used to generate subsections, subheadings, and tables. The selected articles were arranged in subheadings like functionality of plant proteins, mechanism of plasma generation, food application of cold plasma treatment, and structural modifications of proteins.

## 3. Results and Discussions

### 3.1. Literature Search

**Figure 1:**
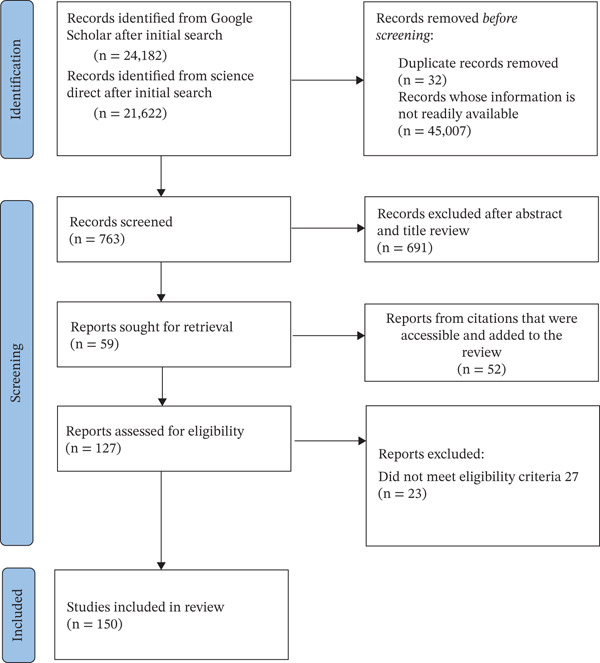
PRISMA 2020 flow diagram of data collection and information search for systematic reviews.

The first literature search generated a total of 45,802 articles, out of which, 24,182 were from Google Scholar and 21,622 from Science Direct. After applying advanced search filters, the results were reduced to 763 and 91 articles, respectively. Afterwards, 32 duplicate records were also eliminated, and an additional 52 relevant articles were found through the reference lists of preselected studies. In total, 150 articles met the eligibility criteria and were included in the review. The overall literature selection process is presented in the PRISMA flow diagram (Figure 1). Out of the studies that were evaluated and used, 75 addressed the utilization of cold plasma for protein modification, 19 focused on their structural changes and the mechanisms involved, and 56 examined their functional roles and applications in the food system.

### 3.2. Core Concept of Cold Plasma Generation

Plasma generation basically entails the creation of ionized gases through electrical charges. It can be broadly classified based on operating pressure into atmospheric plasma and low‐pressure plasma within which various discharge configurations such as radio frequency (RF), dielectric barrier discharge (DBD), and microwave discharge (MWD) are employed [22].

Atmospheric pressure plasma jet (APPJ) involves the creation of atmospheric pressure, usually using DBD or related configuration, which allows for the direct treatment of materials without requiring vacuum system. These plasmas are abundant in reactive species and are employed in sterilization, surface modification, and other related food applications [22]. It is characterized by a flowing gas stream that is mostly either argon, helium, nitrogen, or air. The gas passes through a discharge zone formed between two centric coplanar electrodes that get excited by a RF [23]. The reactive species are generated with pressure at or near atmospheric pressure, usually using electrical discharges like the DBD or capillary plasma electrode discharges. The strength lies in its ability to create large‐volume, nonequilibrium plasmas without the need for vacuum system [24]. According to [25], the resulting plasma emerges as a visible jet that enables the localized treatment of surfaces without the need for samples to be placed in between electrodes.

Low pressure/vacuum plasma is typically created in vacuum chamber or reduced‐pressure environment that allows for more uniform plasma and precise control over the chemistry of plasma but needs more sophisticated equipment. It employs methods like direct current glow discharges, inductively or capacitively coupled radio frequencies discharges (RFD), and MWD, which permit precise control over the properties of plasma and are widely employed in microelectronics and material processing [26]. The choice of plasma creation is influenced by the desired characteristic of plasma including temperature, density, equilibrium state, and the intended use or application [27]. Despite the fact that each of these methods provides their unique advantages, DBD and atmospheric plasma are easier and more scalable for use in the food industry, whereas low‐pressure plasma gives a higher control of specialized applications [28, 29].

The underlying mechanism for cold plasma technology involves a direct reaction between the species generated by plasma and biomolecular targets like plant proteins. The species tend to interact with the surface of the plant protein structure and/or any other biomolecule without causing any damage or heating effect. It usually begins with electron transfer or hydrogen abstraction resulting in the breakage of bonds and the formation of new functional groups [30, 31]. This leads to a slight change in the structure through unfolding and partial oxidation of the amino acids. The effect generally depends on the type of biomolecule, the nature and dose of reactive species, and the plasma operating conditions such as feed gas and exposure time [31]. This technique helps to increase the number of exposed active sites of the plant protein molecules, thereby improving their ability to bind water, dissolve, and foam due to structural rearrangement at molecular level while maintaining their nutritional quality. Some innovative techniques such as molecular dynamics simulation and machine learning are increasingly used to predict and understand these interactions at the atomic level, revealing that sulfur‐containing and polar amino acids are usually prone to plasma‐induced modifications [32].

According to Gonçalves et al. [33], key species generated during plasma treatment are the ROS, RNS, UV photons, and charged particles including ions and electrons. Reactive oxygen species (ROS) such as hydrogen peroxide, hydroxyl radicals, and superoxides, as well as reactive oxygen and nitrogen species (RONS) such as nitric oxides, nitrites, and nitrates are specifically crucial for the biological and chemical effects, as they are capable of causing modifications in biomolecules, antimicrobial activity, cell signaling, or cell death depending on the context [34]. Ultraviolet (UV) emitted photons by plasma contribute to DNA and protein damage, whereas charged particles and electric fields are capable of directly disrupting the cell membranes and molecular structures [35]. The specific composition and concentration of these reactive species are dependent on plasma, the condition of generation and time of exposure [36].

Plasma interaction with biomolecules like plant protein is primarily through the activities of reactive species such as RONS, which are capable of inducing chemical reactions such as oxidation, hydroxylation, nitrosylation, and carboxylation of amino acids, peptides, proteins, and other micro molecules [30]. These modifications are capable of causing changes in structures of proteins like alteration of the secondary structure, thereby affecting protein function, stability and allergenicity.

**Figure 2:**
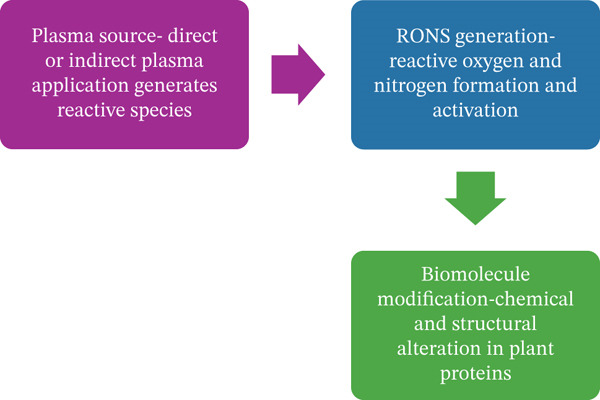
Basic steps involved in cold plasma treatment (source: [38]).

The mechanisms include direct chemical reactions such as abstraction of hydrogen and sulfonation and formation of salt bridges and hydrogen bonds. Other physical effects involved include electroporation when plasma is in direct contact with cells [32]. The specific result is influenced by type of plasma, the nature of biomolecule, and the surrounding environment, with sulfur‐containing and polar amino acids being vulnerable to oxidative modifications (Figure 2). Plasma can also induce modifications in fats or lipids as well as polysaccharides, contributing to their stability and functional properties, which are applicable to the field of food sciences and plasma medicine [37].

**Figure 3:**
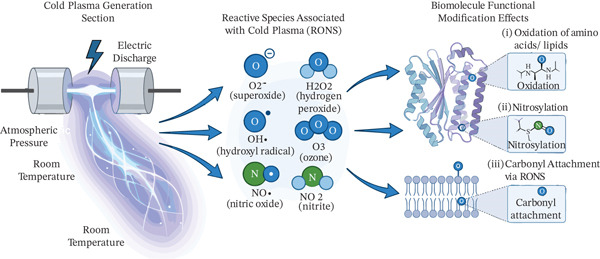
Principles of plasma generation (adapted from [37, 39]).

Overall, the effectiveness of cold plasma technology is largely determined by the type of plasma generated and the reactive species produced, which collectively drive structural and functional modifications in plant protein and other food biomolecules [37] as shown in Figure 3.

#### 3.2.1. Various Discharge Configurations

##### 3.2.1.1. RF

For RF, a mixture of gases, comprising CO_2_ and argon, is employed to generate the plasma, and the resulting ionized energy [40]. It is usually generated by applying alternating electromagnetic field to gaseous medium to produce specific type of plasma and is typically measured in MHz units [41]. A relatively uniform and stable plasma that is characterized by controlled electron energy distribution and moderate gas ionization is produced [42]. Here, the ROS/RNS, including •OH, atomic oxygen (O), ozone (O_3_), and nitrogen‐based species, are generated depending on the composition of the feed gas [43]. Due to its relatively controlled energy input, RF plasma induces mild to moderate oxidative changes in plant proteins. These modifications primarily affect surface‐exposed amino acid residues, including cysteine, methionine, and tryptophan. The result of these modifications is partial unfolding of protein structures and increased exposure of functional groups [37]. RF plasma treatment has been shown to enhance protein solubility, emulsification, and water‐binding capacity by means of structural disintegration and can also enhance enzymatic accessibility during digestion [44]. The difference between RF and other configurations is it produces controlled and mild effect on protein structure. Nevertheless, excessive exposure may result in a series of undesirable outcomes, including oxidation, protein cross‐linking, and compromised functional quality [45].

##### 3.2.1.2. DBD

The DBD configuration most commonly employed uses one insulating (dielectric) layer between electrodes, which creates a barrier against continual current that leads to self‐pulsing, filamentary discharges [28]. They can create nonthermal plasma efficiently in addition to providing safe applications such as generation of ozone and surface modification [46–48]. DBD plasma is marked by creation of several short‐lived micro discharges known as filaments or under some conditions, homogenous discharges, with electron energies suitable for exciting or dissociating gas molecules [49]. This limits the transfer of discharge, which extinguishes rapidly and restarts with each cycle of voltage that enhances stable operation over a wide range of frequencies and pressures [50]. DBD is applied in industries and sciences including ozone creation, control of pollution, surface treatment, excimer lamps plasma display panels, and soft ionization for analytical procedures [51]. The dynamics of the discharge are influenced by several factors such as type of gas, pressure, configuration of electrode, properties of the dielectric, and driving frequencies, which determine the type of discharge either filamentous or homogenous [49].

In the food industry, the reactive species that are created in the oxidative environment interact with plant food biomolecules, especially proteins, which lead to amino acid oxidation, disruption of the disulfide bonds, and unfolding of the secondary and tertiary structures [37]. This structural modification leads to improved digestibility due to the enzyme′s ability to access and improve the functional property of the protein [44]. They generate localized high‐density reactive species that could lead to strong surface modifications that can be sometimes undesirable [52]. As such, DBD plasma is a versatile and efficient nonthermal technology that enables controlled generation of reactive species resulting in beneficial structural and functional modifications plant protein [53].

##### 3.2.1.3. MWD

In the generation of MWD plasma, high‐frequency electromagnetic waves operating within gigahertz (GHz) range are used to produce high‐density plasma that is characterized by elevated electron energy levels [54]. It produces rapid and extensive oxidation of plant proteins. This system often generates a highly energetic plasma environment when compared with RF and DBD configurations [55, 56]. Because of its high energy density, it induces more rapid and extensive oxidation reactions in plant proteins, which leads to the disruption of the intramolecular bonds, conformational changes, and improved functionalities. Therefore, it significantly alters the structure of proteins, improving their solubility, hydration properties, and digestibility. These characteristics are highly desirable for developing protein concentrates, isolates, and structured plant‐based foods, such as meat analogues [57]. The technology is also effective in food decontamination, inactivating enzymes, and surface treatments of food packaging materials [44, 58].

### 3.3. Key Reactive Species Involved in Cold Plasma

The key reactive species created by plasma include ions, electrons, free radical ozone, and ROS such as hydroxyl radicals (^*^OH), singlet oxygen (^1^O_2_), superoxide anion (O_2_^*–^), hydrogen peroxide (H_2_O_2_), and RONS like NO, NO^−^_2_, and NO^−^_3_ [59]. These molecules are highly energetic and are formed when gases like oxygen or nitrogen are energized by electricity. The type of species and concentration depends on several factors and environmental conditions, with gases producing different amount of each of these species [60]. UV photons and charged particles are also generated by plasma, which contribute to the overall reactivity and biological effects of plasma [61]. These reactive species and physical agents are capable of penetrating biological tissues and interact with biomolecules such as plant proteins, leading to an array of effects from signaling of cells to cytotoxicity [62]. Collectively, they are chemically active and constitute the primary drivers of plasma‐induced transformations in plant protein modification and other food macromolecules.

### 3.4. Factors Affecting Treatment Efficiency

Plasma treatment efficiency is greatly influenced by several parameters including type of gas, exposure time, power input, and matrix of the targeted material as in plant proteins and other biomolecules. The choice of gas, for instance, air, oxygen, and argon affects the concentrations of the generated species. This directly affects the extent of modification and degradation [63]. This is because they tend to control the ionization pathway as well as energy transfer in the medium, which has influence on species lifetime and diffusion rate [64]. The exposure time is critical because it determines the extent of modification with longer exposure times leading to increased levels of degradation or modification and vice versa. However, this may lead to diminishing returns or undesired side effects [65].

The availability of energy for plasma generation and reactive species production is influenced by input power; higher power input generally increases the efficiency of treatment but can also lead to an increase in energy consumption and potential damage of some sensitive biomolecules or materials [66]. The nature of the targeted matrix including that of plant proteins and/or other materials can have effect on the extent of interaction of the generated plasma species about modification in the structural matrix. Factors such as temperature and concentration play vital roles in this interaction and the resultant modifications [67]. Other operational indices like rheology, humidity, and reactor design can further regulate the treatment outcomes by affecting the mass transfer and the residence time of the reactive species [32]. Therefore, in order to achieve desired modification outcomes, careful adjustment of processing variables is essential to balancing treatment effectiveness with the integrity of the food material [67].

### 3.5. Structural Modifications of Plant Proteins by Cold Plasma

Cold plasma treatment exerts significant structural changes in plant proteins, improving their functional properties for food applications. The technique usually transforms the *α*‐helices and random coils into *β*‐sheets and *β*‐turns at the secondary structure level as reported for some seeds and legume proteins, causing an increase in protein solubility, emulsification, and foaming capacities [68]. These changes are caused by oxidation and etching effects from the reactive species generated plasma that expose internal hydrophobic groups, decrease particle sizes, and improve the unfolding of proteins or aggregation based on the intensity of the treatment [69–71]. The modification of tertiary structures includes intensity of fluorescence, decreased surface roughness, and altered zeta potential, a reflection of changes in the folding of protein and surface properties [72]. Cold plasma is capable of inducing cross‐linking, including disulfide bonds, dityrosine formation, and increased carbonyl content, further stabilizing or modifying the protein networks [73]. Although exposure to moderate plasma usually enhances the functionality of protein and reduces allergenicity, too much exposure may lead to aggregation and loss of desirable properties, emphasizing the significance of process optimization parameters [5, 6]. This means that the extent and nature of this structural rearrangement determines whether cold plasma enhances protein functionality or promotes undesirable aggregation.

### 3.6. Alterations in Hydrophobicity, Surface Charge, and Free Sulfhydryl Groups

Cold plasma treatment can lead to important alteration of hydrophobicity, surface charges, and free sulfhydryl groups of proteins. The exposure to plasma usually increases the hydrophobicity through unfolding structure of protein and exposure of the hydrophobic amino acid residues in the protein of some pulses, cereals, and legumes [74]. Surface charge usually measured in zeta potential is capable of increasing as a result of accumulation of negatively charged products on the protein surface, which improves electrostatic repulsion and can improve the emulsion stability [75]. Again, in the free sulfhydryl groups, plasma is capable of increasing or decreasing their composition depending on the protein and condition of treatment. Moderate exposure to plasma may lead to an increase in free sulfhydryl content as a result of the unfolding of protein, whereas high exposure or prolonged treatment usually decreases the content of free sulfhydryl groups. This is due to their oxidization to produce disulfide bonds, supporting the aggregation of protein and structural stabilization [76]. These modifications generally improve the solubility, emulsification, and foaming capacities of proteins. However, too much treatment may cause aggregation and loss of functionality [77]. Functional behaviors of most plant proteins are largely governed by changes in their molecular attributes and serve as key indicators of many plasma‐induced modifications [78].

### 3.7. Cross‐Linking and Unfolding Mechanism

In cold plasma technology, the reactive species have been shown to facilitate bond formation in plant proteins due to their reactions with the amino acid moiety, thereby disrupting the noncovalent bonds and forming dityrosine bridge. This process ultimately leads to protein clumping and structural integration and further stabilizing and/or modifying the protein networks [79]. At the same time, plasma treatment may cause the unfolding of protein through the breakage of hydrogen bonds to expose the hydrophobic and sulfhydryl groups, which improves the hydrophobicity of the surface and facilitates further cross‐linking through hydrophobic interactions and formation of disulfide bonds [80].

The balance between unfolding and cross‐linking is influenced by plasma parameters (voltage, time of exposure, and type of protein). These changes in structure could decrease denaturation temperature, improve solubility, and gelation and emulsifying properties, as unfolded proteins are more reactive and capable of forming stronger network [81]. Therefore, the interplay between protein unfolding and crosslinking determines the balance between increased molecular flexibility and network formation during plasma treatment.

### 3.8. Protein Aggregation and Denaturation Patterns

Protein aggregation and denaturation are both products of cold plasma treatment, with patterns influenced by the type of proteins, parameters of plasma, and the reactive species generated. Aggregation is usually supported by the production of new disulfide bonds and oxidative cross‐linking as has been seen in plant proteins and even serum where plasma‐induced ROS/RNS drive self‐assembly and the production of larger or more closely packed aggregates [81]. Denaturation results from the unfolding of secondary and tertiary structures, exposition of hydrophobic groups, and interruption of hydrogen bonds, which can be identified through the changes in fluorescence, circular dichroism, and FTIR spectra [68].

The pattern of aggregation varies, with the initial process leading to the formation of larger aggregates, followed by mutilation into smaller, distorted aggregates with prolonged exposure [81]. In some cases, the treatment with plasma may lead to increase in solubility and functional properties of proteins by partly denaturing them and redistributing the subunits of proteins, whereas excess treatment may result in irreversible aggregation and loss of function [82]. In microbial systems, cold plasma could lead to protein denaturation and aggregation as part of its antimicrobial activity, usually through oxidative stress and the formation of misfolded or ubiquitinated proteins [83]. Within the food system, denaturation and aggregation are interconnected outcomes of plasma exposure. These two processes significantly affect the final physicochemical and functional properties of most plant protein [81].

### 3.9. Functional Properties Improved by Cold Plasma

Cold plasma treatment is vital in the improvement of functional properties of proteins of both plant and animal origin, making it more suited for food application. Some vital enhancements include increased solubility, WHC, emulsion stability, foaming capacity, and gelling properties of several protein sources like peanut, millet, sunflower, soy, and dairy proteins [69–71]. These improved functional properties are linked to some structural modifications like unfolding of proteins, enhanced surface hydrophobicity, altered secondary structure, and exposition of internal groups, which facilitates better interaction with water and other food components [84].

#### 3.9.1. Solubility

Cold plasma treatment is crucial in the enhancement of solubility, hydration, and dispersity of proteins of plant origins through the induction of structural changes that could facilitate their interactions with water. For instance, the solubility of peanut protein isolate is increased by up to 12.17%, which is linked to a rougher, more loosely bound surface, greater exposure of hydrophilic groups, and a shift from compact unfolded protein structure [85]. Soy protein isolate has shown similar improvements, rising from 46% to 66% solubility and dispersion stability increased as a result of particle size, hydrophobicity of the surface, and unfolding of protein that exposed more water‐binding sites [86, 87].

These effects are attributed to the ability of plasma to oxidize and etch protein surfaces, thereby increasing the amount of hydrophilic groups such as the ‐COOH and ‐OH groups and improving the flexibility and hydration properties of the proteins [69–71]. Cold plasma has been able to support the transition of weakly bound water to strongly bound water in high‐denatured peanut protein, further enhancing hydration [88]. Overall, improved solubility has been established as one of the earliest manifestations of plasma‐induced structural modifications and often serves as a marker for enhanced functionality.

#### 3.9.2. Emulsifying Properties

According to Teng et al. [89], there have been reported improvements in the ability of proteins to form emulsions with cold plasma treatment through the enhancement of interfacial activity and the stability of emulsions. This is achieved through the modification of structures including unfolding of proteins, increased surface hydrophobicity, and the exposure of functional groups, which supports improved adsorption at the oil–water interface and the production of robust interfacial films [89]. In grass pea and coconut globulin proteins for instance, moderate cold plasma generation could lead to smaller, more homogenous formations of dense, elastic interfacial layers [90].

Similarly, proteins from cold plasma modification have shown higher emulsifying properties, decreased interfacial tension, and higher stability against creaming and aggregation, resulting in more homogenous and stable emulsion [91]. These improvements are linked to improved surface charge, enhanced solubility and increased shelf life of emulsions [92]. Generally, cold plasma provides nonthermal emulsion formation and stability in food systems making it a better alternative. In many food products, especially those involving the use of plant proteins, enhancing the emulsifying properties during processing helps stabilize the interface between oil and water [93, 94]. This is important in enhancing emulsion stability, texture, mouthfeel, and shelf life. In products like beverages, salad dressings, sauces, and meat analogues, the stability of dispersions during production is paramount for consumer acceptance as well as quality of the end products [95, 96]. As such, CP treatment has been shown to enhance interfacial behavior and emulsion stability, thereby expanding the application of plant proteins in complex food formulations.

#### 3.9.3. Foaming Ability and Stability

Foaming capacity and stability of plant proteins are significantly improved by CP treatment. It is highly important for bakery and beverage production. Studies on peas and zein protein isolates revealed that deamination and glycosylation assisted by plasma improve foamability by up to 223% and foaming stability by up to 100% for the pea isolate due to increased hydrophobicity, unfolding of proteins, and interfacial adsorption in the air–water interface [97]. These changes in structure will expose the hydrophobic groups and increase flexibility of protein molecules, thereby enabling them to create thicker, elastic interfacial layers that stabilize foams and prevent rapid collapse or damage [98].

Enhancements in foaming properties are valuable in the creation of a stable, aerated texture in baked foods and beverages that promotes the development of plant‐based, low energy, and hypoallergenic‐foamed foods [99]. Furthermore, CP is a nonpolluting, nonthermal technology, which makes it suited for preserving the nutritional and sensory qualities of food products while also improving their functional properties [97]. Improvements in the foaming performance, especially for aerated foods that require stable and resilient form structures, help to expand the suitability of plasma modified proteins for food product development [100].

#### 3.9.4. Gelation

Gel network structures are improved and modified via cold plasma treatment. This is due to aggregation and increased cross‐linking that results in enhanced gelation properties across both plant and animal proteins [101]. Cold plasma allows production of gels at lower temperatures of 70°C–90°C instead of higher temperatures > 95°C in pea proteins. This produces gels of interconnected networks, homogenous, higher mechanical strength, viscoelastic, and WHC [11, 12]. These properties are triggered by the formation of fibrillar aggregates, increased surface hydrophobicity exposure of the sulfhydryl groups, and support of hydrophobic interactions, disulfide bonds, and hydrogen bonding within the matrix of the gel [102].

In a similar manner, animal proteins such as surimi and myofibrillar proteins are improved by CP technology, which helps to create a denser, stronger gel network with superior WHC and mechanical properties [103]. Therefore, plasma provides a versatile, nonthermal approach to engineering gel network structures.

In the food industry, the ability of plant proteins to form gels is of particular significance during food formulation. This is because protein gels are capable of constructing three‐dimensional networks that effectively entrap water, lipids, and other food components [104]. This property contributes to the development of desirable qualities such as texture, structural integrity, WHC, sensory perception, and storage stability in the food system [105]. In products like yogurt, desserts, plant‐based meat analogues, and dairy alternatives, these properties are particularly important because the gel structure largely determines product quality and consumer acceptance of these products [106]. Recently, protein gelation is also being used for nutrient encapsulation, fat replacement, and controlled delivery of bioactive compounds in functional foods [107].

In many cases, a reduction in gelation temperature is advantageous. This is because it allows for gel formation under less extreme processing conditions, leading to a reduction in energy requirements and minimized thermal degradation of heat‐sensitive nutrients, flavors, pigments, and bioactive compounds [108]. A reduction in gelation temperature is often associated with protein networks that are more homogenous with enhanced water retention and textural properties and in turn leads to improved functionality and stability of the product. Cold plasma has been shown to influence gel formation, thereby offering potential opportunities for developing protein‐based foods that exhibit enhanced texture, stability, and processing efficiency.

#### 3.9.5. WHC and OHC

Increased WHC and OHC, respectively, are desirable functional properties in many protein‐based foods, especially those of plant sources. However, many of these proteins have less WHC and OHC as compared with their animal counterparts or even carbohydrates‐rich foods such as cereals. This has great implications for food texture and other product quality. For instance, jackfruit seed flour, an excellent source of plant protein in combination with millet flours both treated with CP showed significant rise in WHC from 2.93 to 3.48 g/g and OHC from 1.94 to 2.35 g/g, with increase in solubility and swelling power [109]. These improvements in OHC and WHC are linked changes induced by plasma including increased surface roughness, starch depolymerization, and exposure to hydrophilic and lipophilic groups, which enable the food matrix to trap more water and oil [16].

Improved WHC and OHC promote better texture, water retention, and mouthfeel in food products, making them more tender, cohesive, and less susceptible to dryness or crumbliness, a quality that is of great value in baked foods, snacks, and extruded products [110]. These enhancements induced by plasma promote the development of gluten‐free and high‐fiber foods with improved stability and sensory appeal [111].

### 3.10. Differential Responses of Cereal and Legumes Proteins to Cold Plasma Treatments

Due to their structural characteristics, various plant proteins respond differently to CP treatments [18]. Cereals are mostly rich in prolamins with generally looser structures and are more accessible to reactive species. This facilitates secondary structural alteration and surface residue modification. Legumes on the other hand have dominant 7S/11S globulins that are more resistant and require more intense treatments for comparable functional changes [69–71].

In comparison with other nonthermal methods, for instance, ultrasound, which causes mechanical disruption and pH shifting and results in unfolding through electrostatic repulsion, CP acts mainly through oxidative modification. Cereal proteins usually respond more strongly to oxidative effects of CP than legume proteins that are mostly more influenced by mechanical or pH unfolding [112]. This calls for the need for substrate specific strategies. Both tertiary and quaternary structure of cereal protein exhibit moderate unfolding that tends to improve solubility and emulsification unlike their legume counterparts where much of their quaternary assemble is retained. This leads to functional enhancement unless the method is complemented with other treatments [113–115]. In other words, since not all plant proteins exhibit the same responses to CP, there is need for precision in treatment methodologies, tailored to align with the distinctive structural characteristics inherent to each protein source.

### 3.11. Bioavailability of CP‐Treated Plant Protein‐Based Food

Plant proteins exhibit significant disparities in terms of nutritional value and composition compared with animal proteins due to a variety of factors, including differences in amino acid profile, bioavailability, antinutrient contents, and digestive enzyme accessibility [116]. Several studies have shown enhanced in vitro digestibility of plant proteins such as pea, mung bean, and other legume‐based isolates subjected to cold plasma treatments [55, 56, 117, 118]. This is due to the oxidative modification and partial unfolding of their structures leading to the exposure of the peptide bonds, which makes them more accessible to the digestive enzymes. In their structure, *α*‐helices are usually converted to *β*‐sheets, which increases enzyme accessibility [117]. In addition, many plant proteins exhibit impaired digestibility within the digestive system. This attributable to the presence of antinutritional elements, such as trypsin inhibitors and phytates [119]. In most legumes, they negatively affect protein digestibility either by restricting enzyme access or by creating complexes that decrease protease activity. These substances have been observed to attach to proteins and/or gastrointestinal proteases, thereby reducing their bioavailability and potential nutritional impact. This limits the activity of trypsin, which is one of the important protein digesting enzymes in the gastrointestinal tract [120]. CP treatment helps to inactivate these antinutritional factors through oxidative modification while simultaneously creating a structural unfolding of storage protein′s structure thereby improving enzyme accessibility and proteolytic efficiency.

The generation of RONS during this process has been demonstrated to promote structural alterations, which include the disruption of disulfide bonds and conformational changes in inhibitor proteins. The combined effect of trypsin inhibitor inactivation and protein structural modification improves protein digestibility and enhances bioaccessibility of amino acids and consequently improves their bioavailability for metabolic use in the body [121, 122].

## 4. Application of Cold Plasma in Food Processing

Cold plasma has been found to have different applications in the food industry as a result of its nonthermal, energy‐efficient, and environmentally friendly properties (Table 1). Plasma modified proteins are increasingly employed in plant‐based meat analogues as a result of their ability to enhance vital functional properties, which are important for mimicking the texture and mouthfeel of animal meat [123, 124]. These modifications equally promote the development of plant‐based substitutes that are more appealing to consumers through improvements in sensory and nutritional qualities, while keeping a sustainable and eco‐friendly production process [125]. Treatment with plasma has been considered as a “green” technology, employing little chemical and energy, and can also reduce antinutritional factors and allergens in plant proteins, thereby further making them suitable for food applications [73].

**Table 1:** Application of cold plasma techniques and their key benefits.

Application area	Products	Key benefit	Specific effect	Consideration	References
Plant‐based meat analogues	Meat analogue from proteins of plant source	Improved functional properties, texture, and mouthfeel	Improved solubility	As a result of minimal addition of chemicals, heat, or energy usage, it is termed “green” technology	[73, 125, 123]
			Enhance emulsification		
			Increased gelling and foaming capacities		
			Decreased antinutrients factors		
			Reduce allergenicity		
Replacement for plant‐based dairy products	Vegan cheese, plant‐based milk from sesame	Enhanced nutritional, functional, and sensory characteristics as well as promotes consumer acceptance	Increased water‐holding capacity (WHC)	Total allergen may need additional treatment conditions or parameters	[60, 126, 127, 124]
			Increased oil‐holding capacity (OHC)		
			Improved solubility and emulsifying capacity		
			Improve foaming capacity		
			Increased protein and calcium content		
			Decreased fat composition		
			Removes beany flavor in legumes		
			Reduced antinutrient including phytates and trypsin inhibitors		
			Increased antioxidant activity		
Bakery and confectionery	Bread, flours (horse gram), starches, edible films	Guarantee food safety; prolonged shelf life; improved ingredient	Sterilization	May reduce moisture content and lead to bread hardness, hence the need for optimization of treatment parameter to preserve texture	[128–130]
			Enhanced nutritional and functional properties of flours		
			Decreased antinutritional factors		
			Enhancement of dough characteristics		
			Improvement of physicochemical, mechanical, and barrier properties		
Functional beverages and protein‐enriched foods	Protein‐based beverages, whey beverages with prebiotics, protein‐enriched foods	Modification of protein structures; enhanced functional and nutritional properties; heat or chemicals are not required	Increased solubility of proteins	Efficiency is influenced by type of protein, treatment conditions including time, intensity, and food matrix	[5, 6, 37, 131, 132]
			Enhanced emulsification and gelling ability		
			Improved antioxidant activity		
			Increased bioactive compound		
			Decreased allergens		
			Improved digestibility		
			Enhanced foaming and water‐holding ability		
			Producing surfactant‐free, clean‐label products		

Cold plasma technology is employed in plant‐based dairy substitutes including milk, yoghurt, and cheese to enhance their nutrients, functionality and sensory properties. In vegan cheese, CP treatment of pea protein led to enhanced OHC and WHC, solubility, emulsifying ability, and foamability, leading to a product with increased protein and calcium content, reduced fat, and increased consumer acceptance in comparison with commercial vegan cheeses [126]. In plant‐based dairy products such as sesame and soy milk, CP can effectively reduce unwanted enzymes such as lipoxygenase that causes beany flavor in legumes and antinutrients including phytates and trypsin inhibitors, while keeping heat‐sensitive nutrients and enhancing their antioxidant activities [127]. Cold plasma also imposes advantageous structural changes in plant proteins, rendering them more suited for dairy substitute applications by enhancing their processing and functional properties [124]. However, although the method can decrease some allergens and antinutritional factors, total elimination may need further optimization of treatment conditions [60].

Cold plasma has emerged as a viable technology in bakery and confectionery applications, primarily because of its ability to promote food safety and enhance ingredient functionality without heat application. In bread production, cold atmospheric plasma treatment has led to total elimination of microbes including bacteria, yeast, and molds, significantly leading to improved shelf life. Additionally, it can also decrease moisture content and improve bread hardness, an indication for further optimization to maintain good texture [130]. For flours including horse gram, treatment with CP will improve nutritional and functional properties, reduce antinutrient, and improve dough characteristics, thereby making them suitable for use in bakery and confectionery products [128]. Furthermore, it is employed in the modification of starches and edible films to improve their physicochemical, mechanical, and barrier properties, which can promote the quality and shelf life of baked foods as well as confections [129].

Due to its ability to cause structural changes in plant proteins, the technology finds ready application in the development of plant‐based protein enriched functional beverages affecting many desirable properties without any requirement for heat or chemical application. In plant‐based protein‐enriched beverages, treatment with CP enhances these properties in the product by making it easier to form a stable, appealing drink and food even with an increased protein content [37]. In a similar manner, animal protein beverages like whey beverages with added prebiotics (xylooligosaccharides) increased antioxidant activity and bioactive compound content upon treatment with CP while preserving sensory qualities and reducing loss induced by heat in comparison with other traditional pasteurization [132]. It improves the technofunctional properties, which are vital for texture and mouthfeel [37]. The technology also ensures the production of surfactant‐free protein suspensions that are valuable for the formulation of clean‐label beverages [131]. These diverse applications illustrate how CP may contribute to both product quality and sustainability across the contemporary food systems.

## 5. Comparative Perspective With Other Protein Modification Techniques and Limitations

As shown in Table 2, thermal and CP treatments both modify the functionality and structure of proteins with different effects. However, the typical industrial thermal treatments may lead to excessive unfolding, aggregation, and irregular cross‐linking of proteins. This impacts a negative effect on the digestibility and functional properties, in addition to a possible reduction of its nutritional quality as well as allergenicity [5, 6]. However, moderate heat can lead to partial unfolding of plant protein, which can yield desirable functional properties, and hence, making this method a double‐edged sword.

**Table 2:** Comparative analysis of protein modification techniques.

Aspect	Cold plasma	Thermal treatment	Pulse electric field	Enzymatic hydrolysis	Fermentation	Ultrasound	High pressure processing	References
Nature	Does not require heat or addition of chemicals	Requires heat	Does not require heat uses high voltage pulses, may require aqueous medium	Bioprocessing	Bioprocessing	It does not require heat	It does not require heat	[5, 6, 37]
Mechanism	Involves the use of reactive species (ROS and RNS)	Protein denaturation is induced by heat	Induces electroporation of cell membrane and protein structure	Peptide bonds broken through actions of enzymes	Peptide bonds broken through actions of microorganisms	It leads to the creation of cavity and mechanical shear	Interruption of noncovalent interactions	[5, 6, 18, 133, 134]
Temperature	Minimum	High	Low to moderate	Ambient to moderate	Controlled microbial inoculation and it is dependent on the species used	Ambient	Ambient	[11, 12]
Chemical usage	Does not require use of chemicals	Does not require the use of chemicals	Does not require the use of chemicals	Only enzymes are required	Only microorganisms are required	Does not require the use of chemicals	Does not require the use of chemicals	[18]
Protein structure	Unfolding of protein structures are controlled and modification of structures are targeted	Leads to excess unfolding of protein, protein aggregation, and irregular cross‐linking	Cause partial unfolding, aggregation and changes in secondary and tertiary structure depending on the field strength and treatment time	Peptide bond cleavage	Peptide bond cleavage	Disruption of secondary and tertiary structures of proteins	Change in conformation	[5, 6, 134]
Solubility	Improved solubility	Reduced aggregation	Could enhance or reduce depending on the type of protein	Enhanced	Enhanced	Enhanced	Inconsistent	[11, 12, 18]
Functional properties	Enhanced emulsification, foaming, water‐ and oil‐holding capacities	Detrimental effects	Enhanced	Enhanced by bioactive peptides	Improved by metabolites	Enhanced	Enhanced	[5, 6, 37]
Digestibility	Increased protein digestibility	Detrimental to protein digestibility	Increased	Significantly increased	Increased	Inconsistent	Improved	[5, 6, 86, 87]
Nutritional quality	Retained	Loss of nutrients	Retained	Release of bioactive peptides	More bioactive compounds are released	Retained	Retained	[5, 6, 86, 87]
Allergenicity	Effectively decreased as a result of breakdown of epitopes	Inconsistent	Partial reduction	Minimal reduction	Inconsistent reduction	No evidence on effects on allergenicity	Poor effect	[18, 93, 94, 133]
Sensory properties	Preserved	May lead to alteration of sensory attributes	Minimal impact	Inconsistent	Inconsistent with possibilities of change in flavor	Retained	Retained	[113–115]
Environmental impact	Environmentally friendly	High energy consumption	Environmentally friendly	Environmentally friendly	Environmentally friendly	Environmentally friendly	Environmentally friendly	[5, 6]
Processing time	Somewhat short	Inconsistent	Very short	From minimal to long	Time consuming ranging from hours to days	Somewhat short or minimum	Short time	[135]
Synergistic effect	Enhanced when combined with ultrasound or enzymatic treatment; enhances the efficiency of enzymatic methods when used as pretreatment	N/A	Increased when combined with mild heat, pH shift, or enzymes	Increased with cold plasma treatments	Increased with cold plasma treatments	Improved when combined with cold plasma	Can combine with other methods to give improved result	[73, 134–136]
Clean label compatibility	Excellent due to nonaddition of additives	Good	Aligns well with clean label since no chemical preservative or additives are required	Moderate with enzyme declaration	Moderate with products of fermentation	Outstanding	Outstanding	[18]
Limitation	Complex mechanisms; technology still in early‐stage of development; requires optimization; scale‐up challenges; may lead to overoxidation if treatment time is long; uniformity in complex matrices	Loss of nutrient; uncontrolled aggregation and modification	High cost of energy required	Access to appropriate substrate is difficult; enzymes are usually expensive; the need for enzyme specificity	Tedious and time consuming; requires controlled conditions; products and metabolites may be inconsistent	Reduction of allergens is limited; needs to be optimized	Less targeted modification; high cost of equipment; effects on allergens are limited	[5, 6, 113–115, 133, 135]

Conversely, CP, which has been designated as a nonthermal technology, uses reactive species to create interaction at the surface level. Modifications occur at the surface residues, and reactive sites cause mild cross linking that does not often lead to a full irreversible denaturation unlike thermal treatment. The temperature is usually very minimal and helps to create some targeted modifications including enhanced solubility, OHC and WHC, improved foaming and emulsification properties, and decreased allergenicity, while preserving the sensory and nutritional qualities of the protein [113–115].

In addition, CP, enzymatic hydrolysis, and fermentation are used for modifications of plant proteins but with different mechanisms and different outcomes with no negative impacts on the nutritional quality through the minimum use of temperature and chemicals [18]. Compared with enzymatic hydrolysis and fermentation, CP reduces allergenicity through the breaking down of specific epitopes of plant proteins (Table 2). Several studies showed that CP achieved reductions in allergenic potentials in foods compared with enzymatic hydrolysis, and with greater effects when combined with enzymatic treatment [93, 94]. Enzymatic hydrolysis and fermentation, on the other hand, both bioprocessing methods that employ enzymes and microorganisms, respectively, to break down peptide bonds to release bioactive peptides and promote digestibility and functionality. However, their efficacy can be limited by access to substrates and reactions [135]. More importantly, pretreatment with CP is capable of improving the efficiency of enzymatic hydrolysis through the unfolding of proteins and exposition of more sites for enzymatic activities, leading to higher degrees of hydrolysis and enhanced solubility in comparison with just enzymatic hydrolysis alone [73]. The combination of CP with enzymatic or fermentation methods may produce synergistic improvements in the modification and functionality of proteins as well as reduction of protein allergenicity [135].

Cold plasma, ultrasound and HPP are all nonthermal processing technologies employed in the modification of proteins and in food processing, each with its own distinct mechanism and effects [5, 6]. For instance, ultrasound treatment often leads to alterations of secondary and tertiary structures, as well as mechanical shear to unfold proteins. However, this treatment can synergistically improve the effects of CP when used in combination, thereby leading to greater modifications of protein activity and structure than either method alone [134].

HPP alters the conformation of proteins through the disruption of noncovalent interactions, which can enhance digestibility and functional properties, but may not usually achieve the same level of targeted modification or reduction of allergenicity compared with cold plasma [133]. Studies have indicated that the combination of CP and HPP or ultrasound treatment can improve protein modification, retain nutrients, and product quality in comparison with single treatment [136]. All three methods are seen as eco‐friendly alternatives to traditional thermal processing, with CP and ultrasound providing most advantages in the preservation of heat‐sensitive nutrients and improving functionality [5, 6]. The choice among these three nonthermal alternatives is influenced by desired protein modification, food matrix, and objectives of processing.

Cold plasma provides several advantages over conventional protein modification methods including thermal and chemical treatments. As a nonthermal, nontoxic, and chemical free technology, CP can improve the functionality such as solubility, emulsifying WHC, OHC, and foaming properties of proteins, improve digestibility, and reduce allergenicity while preserving the nutritional quality of foods in comparison with the thermal or chemical processing methods. Thermal and chemical processing methods often result in increased protein unfolding, aggregation, and nutrient loss [37]. Cold plasma equally enables more targeted and tunable modification of proteins by introducing new functional groups and altering the structure of protein without the addition of food additives, making them an alternative for clean‐label and minimally processed foods [18]. Overall, each modification technique possesses its own unique advantages. However, the distinctive ability of CPs to induce targeted alterations without the need for excessive heat renders it particularly suitable for applications plant protein products development.

### 5.1. Synergistic Impacts of Cold Plasma With Other Novel Technologies

Integrating cold plasma with other novel processing technologies represents a shift in direction for food engineering. Although individual nonthermal technologies provide unique structural modifications, their limited operational scope or localized effects often render them not so effective in isolation [137]. Combining these methods produces a synergistic effect, which overcomes the limitations of individual components, thereby facilitating rapid structural transformation and significantly enhancing their technofunctionalities such as solubility, gelling, emulsification, and foaming of plant proteins. For example, plasma may be unable to affect materials below the surface just as ultrasound treatments alone may not be sufficient to eliminate all harmful organisms [138].

Most nonthermal technologies including CP make use of physical, electrical, or chemical methods that function at or close to ambient temperatures [139]. They mostly help to improve structural qualities of food as well as inactivate pathogens and spoilage organisms while preserving nutritional and sensory properties [86, 87]. Ultrasound technology and CP treatments have been shown to be effective in addressing modern processing challenges in the food system [137]. In United States, a process known as waves which help to form microbubbles that collapse rapidly with a localized region of increased pressure and temperature that facilitate the breakdown of cellular structures, enhance the movement of substances within the cell, and improve specific functional properties are then created [140].

One of those synergistic methods is the sonication‐assisted cold plasma (SACP). It is a novel technology that combines the chemical reactivity of plasma‐generated species with the physical forces generated by acoustic cavitation [137]. The synergy helps to improve the movement of reactive molecules into food substrates, enhances the inactivation of microbes and enzymes, and maintains nutritional and sensory properties [137].

In US technology, intense physical forces are generated through the process of acoustic cavitation. This results in the formation of microjets, extreme shear stress, and localized high temperatures [48]. When this is combined with CP, it tends to act as a mechanical catalyst, which facilitates the disruption of large, insoluble plant protein aggregates, thereby unfolding the compact globular matrices that are typical of plant protein such as soy, pea, and faba bean proteins [52]. The amino acids get exposed and are accessible to the reactive species generated; simultaneously, the primary and secondary structures of proteins are then altered. Therefore, the combination of mechanical breakdown and chemical modification results in increased plant protein solubility.

In PEF technology, ultrashort electrical pulses at elevated field strengths are employed to induce electroporation and polarization within a protein solution [141]. In combination with CP, they produce powerful dual action where the PEF helps to polarize and stretch the protein molecule and disrupts the native electrostatic interactions holding the tertiary structures together. This makes it easy for reactive species generated to chemically attack the exposed peptide links and sulfur‐containing amino acids [7, 37]. This is especially important for gelation where proteins form electrostatic hydrogels at low temperatures.

Enzymatic hydrolysis is a nonthermal food processing method where specific endo‐ and exo‐proteases cleave peptide bonds and break down large native proteins into functional bioactive peptides [142]. For legumes such as soy glycinin or pea legumin that are high in globular proteins, they exhibit rigid, compact tertiary structures, which often result in their key cleavage sites being embedded deep within their hydrophobic cores [135]. This quality places a limitation on the efficiency of the enzymes to act upon them. To overcome this, CP is applied as a pretreatment where its RONS creates an intense microbombardment on the protein surface to induce a localized structural partial unfolding without fully denaturing the protein [93, 94]. Therefore, tightly coiled alpha helices are transformed into more accessible random coils [18, 23]. Enzymes such as papain bind rapidly to the target substrates and drastically accelerate the degree of hydrolysis and improve their solubility.

Aromatic amino acid residues such as tyrosine and tryptophan are usually oxidized when ozone is combined with CP. Furthermore, ozone basically acts as a potent oxidizer, and the net surface negative charge is increased through irregular surface cavity created in the process that alters the morphological characteristics of the protein [37, 143]. Overall, integrating CP with other emerging technologies helps to overcome the limitations imposed by individual processing methodologies. Table 3 shows a highlight of some of these synergies with the various impacts on plant proteins as well as their merits and demerits in the food industry.

**Table 3:** Synergistic impacts of cold plasma with other novel technologies on plant protein.

Synergy	Impact	Advantages	Disadvantages	Reference
Cold plasma + ultrasound	Disruption of dense protein aggregates and unfolding of globular proteins	Rapidly improves solubility, foaming and emulsification properties	High‐energy use	[38, 53, 119, 144]
			Risk of localized heat generation if not properly monitored	
	Surface area exposure	Improves process efficiency		
	Modification effect through the action of the generated plasma			
Cold plasma + pulse electric field	Molecular polarization and structural elongation caused by high voltage pulses which result in exposure of electrostatic sites proned to targeted chemical crosslinking through the reactive species created by CP	Operation is completely free from thermal footprint	High‐cost implication	[38, 143]
			Requires pumpable fluids or slurries	
		It helps to minimize bean off flavors especially in legumes		
Cold plasma + microwave	Radiation from microwave relaxes the internal hydrogen bond by dipole rotation which helps facilitate volumetric penetration of CP species	Modification is highly uniform although the sample	Risk of heat induced denaturation is unmonitored	[18, 128, 143]
			Scaling up to large industrial volume is usually a challenge	
		It significantly improves WHC and OHC		
Cold plasma + O_3_	Increased net negative charge and controlled surface carbonylation are induced through the combination of O_3_ and cold plasma′s free radical	Very effective in the modification of surface charges for extreme dispersibility	High risk of over oxidation	[128]
			Requires a complex infrastructure for monitoring and ensuring the gas is sealed	
		Also effective in microbial sanitation		
Cold plasma and enzymatic hydrolysis	CP‐generated RONS physical disruption and unfold tight, globular structures and expose buried peptide bonds for rapid, targeted protease cleavage	Fast in improving degree of hydrolysis of proteins	Requires adequate control over the intensity of the CP to avoid damaging the enzyme	[18, 38, 69–71, 145]
		Generates highly functional bioactive peptides		
		Improved emulsifying ability and stability		
	Side chain oxidation			
		Improved OHC		
		Enhance solubility		

### 5.2. Challenges and Limitations

Despite the many advantages of cold plasma treatment on plant protein functionality as well as the environment, its success is characterized by several limitations especially on process parameters. These parameters have significant impact on the type and concentration of the reactive species generated, as well as the UV radiation and the energized electron, which could lead to variations in the outcome [5, 6, 146]. Enzyme structure and function are also reported to be affected by different active species of plasma and can as well lead to variations [146]. This makes reproducibility a major concern.

The technology although beneficial, is also capable of causing protein and lipid degradation and loss of natural antioxidants caused by the RONS that are generated during the process. This results in reduced nutritional quality, off flavor and also affects sensitive amino acids that could indicate a tradeoff between functionality and nutrient retention [147].

Another important limitation of CP treatment is nonuniform modification, especially with food having thick or complex matrices. For such food, their effectiveness in bulk systems is restricted. This is because the technique is mostly a surface treatment, making penetration shallow as reactive species have short lifespans. In high moisture foods or aqueous protein dispersion, reactive species show poor penetration into the liquid phase, leading to localized effect and reduced uniformity. For low moisture food, there is usually interaction between reactive species and substrates [18, 137].

Again, some of the key issues to be addressed when upscaling this technology include difficulty in optimizing the level of measurable and repeated plasma treatment for plant protein food products as well as ensuring operational safety. In addition, lack of standardized protocols for plasma treatment in plant protein modifications could sometimes induce undesirable odor taste, and color in some kinds of plant proteins compared with others. This could be due to oxidation in lipid‐rich plant proteins such as peanut and moringa seeds due to lipid oxidation, which can also cause discoloration in protein powder, raising regulatory and consumer acceptance concerns. For these reasons, several technical and practical challenges must be addressed before CP can be reliably implemented on an industrial scale.

## 6. Future Perspectives

Cold plasma technology for improving plant protein functionality is still at its early stage of evaluation. Therefore, to advance this technology, it is crucial to optimize the process parameters. Integration with other innovative nonthermal technologies like ultrasound, fermentation, high pressure treatment, and pulse electric field applications could also offer a highly effective means of overcoming the scientific and technological limitations related to the use of food proteins, especially those from plant sources.

Furthermore, although CP is gradually being adopted for protein modifications, its underlying mechanisms are not fully understood. Although ROS/RNS are identified as the primary drivers of structural and functional changes in proteins, the specific pathway of protein oxidation, cross‐linking, and conformational rearrangement is not fully understood. In addition, protein‐specific responses to CP are still unclear as to which proteins are inherently more suitable for its treatment.

Therefore, future research should delve into developing predictive and computational models to anticipate functional outcomes across diverse food systems. Elucidating the mechanistic pathways of CP‐induced modifications at molecular levels will also be useful to this technology. Adopting omics approaches that include proteomics, genomics, and metabolomics to study plasma–protein interactions will also be beneficial to the science of improving plant‐based protein functionality.

## 7. Conclusion

Cold plasma is gradually becoming one of the prominent ways of improving plant‐based protein functionality. Cold plasma has been shown to be an effective, nonthermal approach for enhancing the functionality of plant proteins by making targeted structural modifications. It demonstrated that changes in protein conformation, surface characteristics, and intermolecular interactions, induced by plasma, contribute to enhanced solubility, emulsification, foaming, gelation, and WHC and OHC. Protein modification by cold plasma technology is achieved through RONS, which induce oxidation and cross‐linking as well as conformational changes. These molecular changes directly influence their functional properties that are naturally minimal or absent in some cases, making them measure up with their animal counterparts and other food ingredients. In comparison with conventional techniques, modifications offered by cold plasma are quite distinct from thermal, enzymatic, or fermentation‐based methods. Understanding the pathway of cold plasma mechanism is central to controlling the functional outcome. Besides improvements in protein functionality, the technology also offers other advantages including extended shelf life of plant‐based protein products, reduced antinutrients present, especially in legumes making it highly beneficial in food formulation. Overall, cold plasma is useful in improving protein functionality without compromising nutritional qualities.

**Nomenclature**
COOH: carboxylic group
CP: cold plasma
DBD: dielectric barrier discharge
DNA: deoxyribose nucleic acid
GHz: gigahertz
H_2_O_2_
: hydrogen peroxide
HPP: high‐pressure processing
MWD: microwave discharge
O_3_
: ozone
OH: hydroxyl radical
OHC: oil‐holding capacity
PEF: pulse electric field
RFD: radio frequencies discharges
RNS: reactive nitrogen species
ROS: reactive oxygen species
US: ultrasound
UV: ultraviolet
WHC: water‐holding capacity

## Author Contributions

Aishat O. Adejoh: draft of original copy, assessment, methodology, manuscript writing, review, and editing, data curation, formal analysis, software, and resource. Linda A. Kpomblekou: manuscript writing and editing. Flora G. C. Ekezie: conceptualization, manuscript writing, review, and editing. Titilayo O. Bamidele: manuscript writing, review, and editing. Alberta N. A. Aryee: manuscript writing, review, and editing. John O. Onuh: conceptualization, investigation, funding acquisition, writing of original draft, methodology, validation, visualization, manuscript writing, review, and editing, project administration, supervision, formal analysis, software, and resources.

## Funding

Funding support for the research was provided by the George Washington Carver Agricultural Experiment Station (GWCAES) of Tuskegee University through the USDA/NIFA Evan Allens Program.

## Conflicts of Interest

The authors declare no conflicts of interest.

## Data Availability

The data that support the findings of this study are available from the corresponding author upon reasonable request.
